# Copy Number Alterations in CDKN2A/2B and MTAP Genes Are Associated With Low MEF2C Expression in T-cell Acute Lymphoblastic Leukemia

**DOI:** 10.7759/cureus.32151

**Published:** 2022-12-03

**Authors:** Sarita Kumari, Jay Singh, Mohit Arora, M. Shadab Ali, Avanish K Pandey, Mercilena Benjamin, Jayanth Kumar Palanichamy, Sameer Bakhshi, Imteyaz Qamar, Anita Chopra

**Affiliations:** 1 School of Biotechnology, Gautam Buddha University, Greater Noida, IND; 2 Laboratory Oncology, BRA-IRCH (Dr. BR Ambedkar Institute Rotary Cancer Hospital), All India Institute of Medical Sciences, New Delhi, New Delhi, IND; 3 Biochemistry, All India Institute of Medical Sciences, New Delhi, New Delhi, IND; 4 Pulmonary Medicine and Sleep Disorders, All India Institute of Medical Sciences, New Delhi, New Delhi, IND; 5 Medical Oncology, BRA-IRCH (Dr. BR Ambedkar Institute Rotary Cancer Hospital), All India Institute of Medical Sciences, New Delhi, New Delhi, IND; 6 School of Biotechnology, Gautam Buddha University, Greater Noida, Uttar Pradesh, IND

**Keywords:** copy number alterations, t-all, leukemia, mtap, cdkn2a/2b, mef2c

## Abstract

The molecular heterogeneity of T-cell acute lymphoblastic leukemia (T-ALL) makes this disease complex. Early T-cell precursor ALL (ETP-ALL) is a recognized subtype of T-ALL associated with a high probability of induction failure with conventional therapy. Higher expression of myocyte enhancer factor 2C (*MEF2C*) and the absence of a biallelic deletion (ABD) are the designated markers for the ETP-ALL. Co-deletion of the contiguous genes cyclin-dependent kinase inhibitor 2A/2B (*CDKN2A*/*2B)* and the methylthioadenosine phosphorylase (*MTAP)* cluster, located at 9p21.3, is another common alteration in T-ALL and confers poor response to treatment. We used real-time polymerase chain reaction (PCR) analysis to assess *MEF2C* mRNA expression and ABD status. Copy number alterations (CNAs) in key genes previously reported to be altered in T-ALL were assessed using multiple ligation probe amplification (MLPA). We observed that CNAs in this co-deletion cluster of *CDKN2A/B* and *MTAP* genes exhibited low *MEF2C* expression while ABD was associated with CNA in the Abelson murine leukemia 1 (*ABL1)* gene. Assessment of *MEF2C* expression based on immunophenotype revealed that its association with *CDKN2A/2B* alteration is present in non-immature immunophenotype. Additionally, ABD was associated with copy number alterations of T-cell acute lymphocytic leukemia protein 1 (*TAL1)*, myeloblastosis (*MYB)*, and LIM domain only 2 (*LMO2)* genes in immature immunophenotypes. Further, *STIL::TAL1* fusion was associated with low expression of *MEF2C*. These associations may help explain the difficulties in assessing disease heterogeneity and the prognostic importance of 9p21.3 alterations in T-ALL.

## Introduction

T-cell acute lymphoblastic leukemia (T-ALL) is an aggressive malignancy that can be stratified into various heterogeneous molecular subgroups [1,2]. These subgroups can be recognized by their characteristic immunophenotypes and by distinctive gene expression profiles. Myocyte enhancer factor 2C (*MEF2C*) is an oncogene belonging to the MADS-box family of transcription factors, which consists of four members: *MEF2A, B, C, and D,* and plays a critical role in embryonic development [3,4]. *MEF2C *is expressed at the onset of cardiac-skeletal muscle lineage differentiation, and *MEF2C *knockout mice are embryonically lethal at the E9.5 stage of development [5]. Further, *MEF2C *also plays a crucial role in the normal hematopoietic system, particularly in the production of immature and mature lymphocytes [4]. In the context of its role in pathophysiology, it has been widely reported to be abnormally expressed in immature subsets of T-ALL, particularly in early thymocyte precursor (ETP) ALL, an entity that has been associated with adverse risk characteristics and poor outcomes in T-ALL [6-9]. Studies have shown that *MEF2C *acts as a transcriptional regulator for genes that are expressed in the immature subgroup of T-ALL, including lymphoblastic leukemia-derived sequence 1 (*Lyl1 *) and LIM Domain Only 2 (*LMO2*) [10]. One group characterized another identifier for the immature subgroup, the absence of biallelic deletion (ABD) of the T-cell receptor (TCR) gamma gene locus, representing an early maturation arrest before the onset of T-cell receptor rearrangements [11]. Previously, our study suggested the association between ABD and high *MEF2C *expression [12]. Further, high *MEF2C *expression was associated with poor overall and event-free survival while ABD exhibited no significant association with patient overall and event-free survival. In addition to transcriptional regulators, there are other factors, such as somatic genetic abnormalities, which can function as initiators and/or drivers of leukemia progression [13].

The genetic landscape of T-ALL and its clinical utility along with the established factors is poorly understood and needs to be studied more comprehensively. Targeting transcription factors pharmacologically has proven to be extremely difficult. Therefore, it is important to integrate other ways to target key signaling pathways, such as neurogenic locus notch homolog protein 1 (NOTCH1), anti-apoptotic signaling pathways, and genetic aberrations and clinical risk factors, to improve prognostication in T-cell precursor acute lymphoblastic leukemia [10,14]. In the current study, we attempted to integrate both transcriptional and genetic aspects of disease and established an association between these factors. Copy number alterations (CNAs), which are somatic modifications to the chromosome structure that cause the addition or deletion of copies of certain DNA segments, are common in many cancer types [15,16]. CNAs are prevalent in all forms of leukemia, including T-ALL, particularly the genes involved in transcription, cell cycle control, and T-cell differentiation [15,17]. According to earlier research, the T-ALL genome frequently demonstrates CNAs in NOTCH1 and cyclin-dependent kinase inhibitor 2A/2B (*CDKN2A/2B)* cell cycle regulators. It has been proposed that CNAs might be useful in improving the T-ALL risk stratification. We previously determined the frequency of key CNAs in a cohort of Indian T-ALL patients using Multiplex Ligation-dependent Probe Amplification (MLPA) [18]. Additionally, we determined the association of CNAs with patient prognosis with a comparative analysis of the existing literature. In order to define the clinical utility of CNAs, it is important to determine their association with other molecular alterations and prognostic markers such as immunophenotype and gene expression profile. In the current study, we analyzed data from 88 T-ALL patients to determine the association of key CNAs with poor-risk indicators ABD and *MEF2C *mRNA expression.

## Materials and methods

Patient samples and treatment protocol

We reassessed the data from our previously published studies [12,15] and included patients for whom both *MEF2C *expression level and status of copy number alteration were available. Briefly, *MEF2C *expression and copy number alteration were determined in 88 patients (age two months to 68 years) who were diagnosed with T-lineage ALL. Berlin-Frankfurt-Muenster98 (BFM98) protocol was used to treat adult T-ALL patients (age ≥18 years), and the Indian Childhood Collaborative Leukemia group protocol (ICiCLe) was used to treat pediatric T-ALL patients (age <18 years) [19]. After giving these individuals steroid prophase for the first seven days, the absolute blast count was assessed on day eight to assess their response to prednisolone. Based on the absolute blast count in peripheral blood (PB) on day eight of medication beginning, the patients were divided into two groups: prednisolone good responders (PGR) and prednisolone poor responders (PPR). As stated in our prior paper, postinduction bone marrow (BM) samples were used to determine the minimal residual disease (MRD). Patients were classified as either MRD positive (MRD >0.01%) or MRD negative (MRD <0.01%) [18]. BM and/or PB samples were collected at the Department of Medical Oncology, Dr. BR Ambedkar Institute Rotary Cancer Hospital (BRA-IRCH), All India Institute of Medical Sciences, New Delhi, India. The current study was approved by the institutional ethics committee, following the Declaration of Helsinki. All patients or guardians/parents gave written informed consent to the use of leftover diagnostic material for research purposes.

Immunophenotyping

All patient samples were collected in ethylenediamine tetraacetic acid (EDTA) vials and immunophenotyping was performed using the stain-lyse-wash method. The immature group was defined by the presence of cytoplasmic CD3, CD2, and CD5+/- and the absence of both CD4 and CD8; the cortical group was defined by the presence of a CD1a marker and the mature group was defined by the presence of a CD3 surface expression and positivity for one of the CD4 or CD8 markers. The diagnosis of ETP was based on previously defined criteria: CD5 weak/negative, CD1a negative, CD8 negative, and expression of one or more myeloid and stem cell markers [9].

Patient characteristics

A total of 88 newly diagnosed T-ALL patients were included in the study. Of these 88 newly diagnosed T-ALL samples, *MEF2C *expression was examined at the mRNA level while copy number change and ABD in DNAs from newly diagnosed patients were examined. There were 76 males and 12 females, which is in agreement with previous reports [20]. There were 64 pediatric and 24 adult patients. Mean hemoglobin, leukocyte, and platelet counts were 9.62 ±2.59 g/dL, 104.09 ±13.4 x 10^9^/L, and 92.38 ±14.6 x 10^9^/L, respectively. According to the European Group for the Immunological Characterization of Leukemias (EGIL) classification [2], there were 34 (38.63%) immature, 41 (46.59%) cortical, and 13 (14.77%) mature T-ALL cases. We also assessed the early T-cell precursor (ETP-ALL) immune phenotype in 16/88 cases (18.18%). Taking post-induction assessment into account, the MRD was positive in 17/88 (19.31%) cases and negative in 71/88 (80.68%) cases. For ABD analysis, 25/88 (28.40%) patients were classified in the ABD group and 63/88 (71.59%) patients in the non-ABD group, as previously reported [11].

Measurement of MEF2C expression

Total RNA was isolated from the T-ALL-BM/PB samples using TRIZol (Thermo Fisher Scientific, Waltham, Massachusetts) reagent according to the recommendation of the manufacturer and reverse transcribed to cDNA using RevertAid First Strand cDNA Synthesis Kit (Thermo Fisher Scientific). *MEF2C *expression was measured by real-time PCR using the same primer and probe as used previously (Table 1) [12]. *β-actin*,* ABL1*, and *GPI* genes were used as housekeeping genes to normalize the real-time Ct value of *MEF2C *to calculate the final 2^ΔCt. For *MEF2C *gene expression, 88 patients were divided into two groups, *MEF2C *high and *MEF2C *low, based on median expression level.

**Table 1:** Primer sequences utilized for the assessment of gene expression by quantitative real-time PCR and absence of biallelic deletion PCR: polymerase chain reaction

Gene name	Forward primer	Reverse primer
TCRG-VJ	CATCCTCACTTTCCTGCTTCTTC	CCAAGGTGAATCCCTACATGCT
ANLN	AAATTCTGCCCTTTGCTTGTTT	GAAAGCAACCACAGAGAATATGTAAGTAA
MEF2C	GCGCTGATCATCTTCAAC	CTTTGCCTGCTGATCATT
ABL1	TGGAGATAACACTCTAAGCATAACTAAAGGT	GATGTAGTTGCTTGGGACCCA
GAPDH	GAAGGTGAAGGTCGGAGTCAAC	CAGAGTTAAAAGCAGCCCTGGT
β-actin	TCACCCACACTGTGCCCATCTACGA	CAGCGGAACCGCTCATTGCCAATGG

ABD assessment of TCR gamma chain

Total DNA was extracted using a DNA extraction kit from Thermo Fisher Scientific. The TCR deletion was determined as described previously [11]. The expression level of TCR was quantified using real-time PCR as a fold change of the anillin, actin binding protein (ANLN) gene compared to T cell receptor gamma-V (variable) and J (joining) gene segments (*TCRG-VJ*) expression. Sequences of primers have been given in Table 1 [12]. For ABD assessment, patients were assigned to the ABD group based on the ratio of TCR: *ANLN, *which* *was > 0.5 and a high blast count of approximately >85%, and patients with a change of <0.35 fold were assigned to the non-ABD group [11].

CNA assessment using MLPA reaction and analysis

MLPA reaction method has been previously published [18]. Briefly, copy number changes were detected using the SALSA MLPA probe mix P383-A2 (MRC Holland, The Netherlands). This kit was used to detect changes in signaling (*PTEN*, *NF1*, and *PTPN2*), cell cycle (*CDKN2A*, *CDKN2B*, and *CASP8AP2*), transcription factors (*LEF1 *and *MYB*), and epigenetic regulator genes (*EZH2*, *SUZ12*, and *PHF6*) and in addition to the identification of* STIL::TAL1* and *NUP214::ABL1* gene fusions. All reactions were performed in a thermal cycler with a preheated lid. 100 ng of DNA was diluted in 5 µl of water and heated to 98°C for 5 min, followed by the addition of probe mix, hybridization, and amplification. The amplified product was then subjected to capillary gel electrophoresis on an ABI Genetic Analyzer (Thermo Fisher Scientific). Data analysis was performed using the Coffalyser.Net software (MRC Holland, The Netherlands) according to the manufacturer's recommended protocol. Patients were divided into five groups for each probe set, as no copy number changes (0.80 to 1.20), heterozygous or homozygous deletions (0.40 to 0.65 and 0.00, respectively), and hetero- and homozygous duplication (0.30 to 1.65 or 1.75 to 2.15, respectively) were noted based on the final probe ratio obtained by normalizing the sample signal to the control signal.

Statistical analysis

The nonparametric Mann-Witney U test for continuous variables and Fisher's exact test for categorical variables were used to examine the associations. All analyses were performed using STATA version 20 (StataCorp LLC, College Station, TX), SPSS (IBM Corp., Armonk, NY), and Graphpad version 8 statistical software (Graphstats Technologies Private Limited, Bengaluru, Karnataka, India). The mean of continuous variables among clinicopathological features was described as the mean ± standard deviation (SD). A p-value of 0.05 (two-tailed) was considered statistically significant.

## Results

Association of *MEF2C *expression with copy number alterations

**Figure 1:**
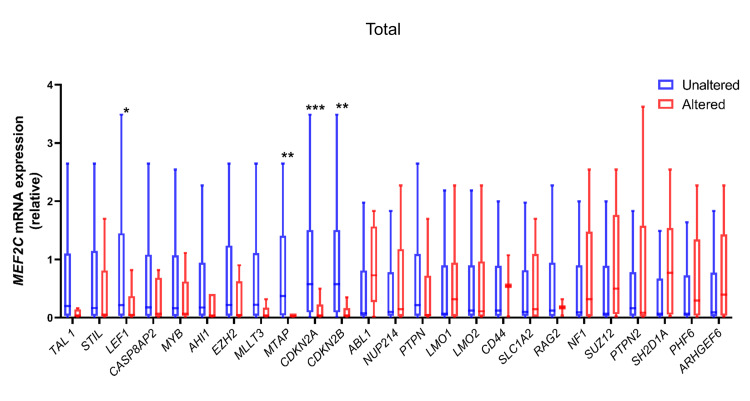
Comparison of MEF2C mRNA expression between T-ALL patient’s groups based on copy number alterations of studied genes The box plot shows Tukey plots without outliers. The Mann-Whitney U test was applied for comparing respective groups based on the alteration status of genes. The level of significance is denoted as ***p<0.001, **p<0.01, *p<0.05.

To determine the association of *MEF2C *expression and ABD with CNA in our patient cohort, we divided patients into two groups, *MEF2C *high and *MEF2C *low, based on the median expression. Higher *MEF2C *expression was associated with lower frequency of CNAs in *CDKN2A *(36.36% vs 84.09%, p<0.0001), *CDKN2B *(25% vs 72.73%, p<0.0001), and *MTAP *(13.64% altered vs 47.73% unaltered, p=0.001) (Figure 1, Table 2) while no association of *MEF2C *expression was observed with CNAs of the genes involved in cell transduction (*PTEN*, *NF1, *and *PTPN2*, p=0.332, 0.783, 1.000, respectively), cell cycle (*CASP8AP2*, p=0.383), transcription factors (*LEF1 *and *MYB*, p=0.132, 0.590, respectively), in epigenetic regulator genes (*EZH2*, *SUZ12, *and *PHF6 *(p=0.493, 0.229, 0.516, respectively), and with known fusion genes (*STIL*, *TAL1*, *NUP214*, *ABL1*, and *RAG2*, p=0.461, 0.118, 1.000, 0.089, 1.000, respectively) and other genes (*CD44*, *SLC1A2*, *AHI*, *MLLT3 *p=1.000, 1.000, 0.314, 0.103, respectively).

**Table 2:** Association of MEF2C expression with the key copy number alterations in total T-ALL cases

	Total number of patients (%)	*MEF2C *low number of patients (%)	*MEF2C *high number of patients (%)	p-value
TAL1				
Unaltered	76(86.36)	35(79.55)	41(93.18)	0.118
Altered	12(13.64)	9(20.45)	3(6.82)	
STIL				
Unaltered	66(75.00)	31(70.45)	35(79.55)	0.461
Altered	22(25.00)	13(29.55)	9(20.45)	
LEF1				
Unaltered	67(76.14)	30(68.18)	37(84.09)	0.132
Altered	21(23.86)	14(31.82)	7(15.91)	
CASP8AP2				
Unaltered	74(84.09)	35(79.55)	39(88.64)	0.383
Altered	14(15.91)	9(20.45)	5(11.36)	
MYB				
Unaltered	71(80.68)	34(77.27)	37(84.09)	0.590
Altered	17(19.32)	10(22.73)	7(15.91)	
AHI1				
Unaltered	78(88.64)	37(84.09)	41(93.18)	0.314
Altered	10(11.36)	7(15.91)	3(6.82)	
EZH2				
Unaltered	60(68.18)	28(63.64)	32(72.73)	0.493
Altered	28(31.82)	16(36.36)	12(27.27)	
MLLT3				
Unaltered	71(80.68)	32(72.73)	39(88.64)	0.103
Altered	17(19.32)	12(27.27)	5(11.36)	
MTAP				
Unaltered	61(69.32)	23(52.27)	38(86.36)	0.001
Altered	27(30.68)	21(47.73)	6(13.64)	
CDKN2A				
Unaltered	35(39.77)	7(15.91)	28(63.64)	<0.0001
Altered	53(60.23)	37(84.09)	16(36.36)	
CDKN2B				
Unaltered	45(51.14)	12(27.27)	33(75.00)	<0.0001
Altered	43(48.86)	32(72.73)	11(25.00)	
ABL1				
Unaltered	78(88.64)	42(95.45)	36(81.82)	0.089
Altered	10(11.36)	2(4.55)	8(18.18)	
NUP214				
Unaltered	63(71.59)	32(72.73)	31(70.45)	1.000
Altered	25(28.41)	12(27.27)	13(29.55)	
PTPN				
Unaltered	65(73.86)	30(68.18)	35(79.55)	0.332
Altered	23(26.14)	14(31.82)	9(20.45)	
LMO1				
Unaltered	55(62.50)	31(70.45)	24(54.55)	0.186
Altered	33(37.50)	13(29.55)	20(45.45)	
LMO2				
Unaltered	80(90.91)	40(90.91)	40(90.91)	1.000
Altered	8(9.09)	4(9.09)	4(9.09)	
CD44				
Unaltered	86(97.73)	43(97.73)	43(97.73)	1.000
Altered	2(2.27)	1(2.27)	1(2.27)	
SLC1A2				
Unaltered	79(89.77)	40(90.91)	39(88.64)	1.000
Altered	9(10.23)	4(9.09)	5(11.36)	
RAG2				
Unaltered	86(97.73)	43(97.73)	43(97.73)	1.000
Altered	2(2.27)	1(2.27)	1(2.27)	
NF1				
Unaltered	72(81.82)	37(84.09)	35(79.55)	0.783
Altered	16(18.18)	7(15.91)	9(20.45)	
SUZ12				
Unaltered	75(85.23)	40(90.91)	35(79.55)	0.229
Altered	13(14.77)	4(9.09)	9(20.45)	
PTPN2				
Unaltered	63(71.59)	31(70.45)	32(72.73)	1.000
Altered	25(28.41)	13(29.55)	12(27.27)	
SH2D1A				
Unaltered	70(79.55)	37(84.09)	33(75.00)	0.429
Altered	18(20.45)	7(15.91)	11(25.00)	
PHF6				
Unaltered	52(59.09)	28(63.64)	24(54.55)	0.516
Altered	36(40.91)	16(36.36)	20(45.45)	
ARHGEF6				
Unaltered	68(77.27)	35(79.55)	33(75.00)	0.800
Altered	20(22.73)	9(20.45)	11(25.00)	

**Figure 2:**
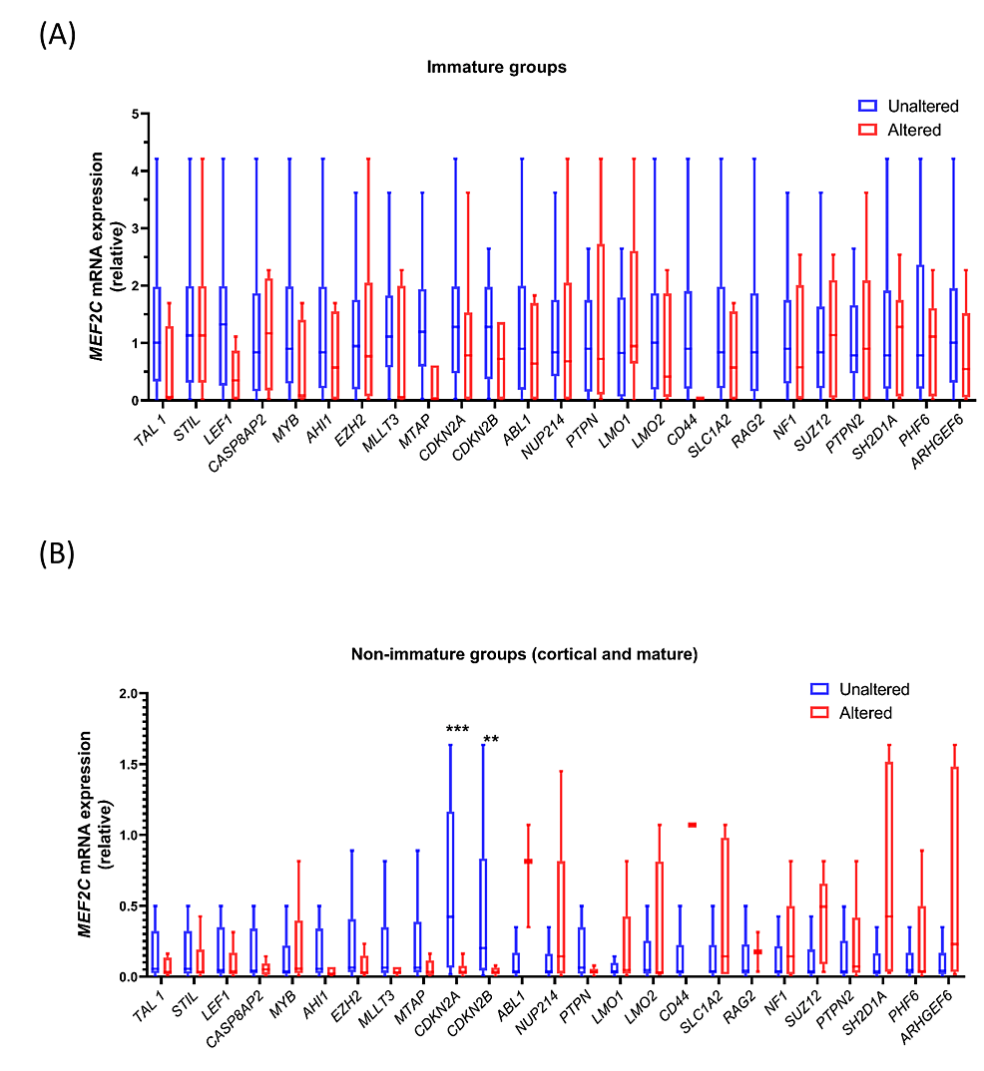
Comparison of MEF2C mRNA expression between patient groups based on copy number alterations of commonly altered genes in T-ALL with (A) immature immunophenotype specific analysis, and (B) non-immature group containing mature and cortical T-ALL immunophenotypes The box plot shows Tukey plots without outliers. The Mann-Whitney U test was applied for comparing respective groups based on the alteration status of genes. The level of significance is denoted as ***p<0.001, **p<0.01, *p<0.05.

We further assessed the association of *MEF2C *expression with CNAs in the immature group separately. This suggested that the *MEF2C *high group was not associated with alterations in the copy number of any of the genes assessed (Figure 2A). In non-immature cases, i.e., cortical and mature, low *MEF2C *expression was observed in cases with CNA in *CDKN2A *(92.59% vs. 55.56%, p=0.004) and *CDKN2B *(81.48% vs. 37.04%, p=0.002) while no association was observed with CNAs of other assessed genes (Figure 2B, Table 3).

**Table 3:** Association of MEF2C expression with the key copy number alterations in non-immature (cortical and mature combined) T-ALL cases

	Total number of patients (%)	*MEF2C *low number of patients (%)	*MEF2C *high number of patients (%)	p-value
TAL1				
Unaltered	46(85.19)	22(81.48)	24(88.89)	0.704
Altered	8(14.81)	5(18.52)	3(11.11)	
STIL				
Unaltered	42(77.78)	20(74.07)	22(81.48)	0.745
Altered	12(22.22)	7(25.93)	5(18.52)	
LEF1				
Unaltered	39(72.22)	19(70.37)	20(74.07)	1.000
Altered	15(27.78)	8(29.63)	7(25.93)	
CASP8AP2				
Unaltered	44(81.48)	22(81.48)	22(81.48)	1.000
Altered	10(18.52)	5(18.52)	5(18.52)	
MYB				
Unaltered	42(77.78)	23(85.19)	19(70.37)	0.327
Altered	12(22.22)	4(14.81)	8(29.63)	
AHI1				
Unaltered	48(88.89)	22(81.48)	26(96.30)	0.192
Altered	6(11.11)	5(18.52)	1(3.70)	
EZH2				
Unaltered	36(66.67)	15(55.56)	21(77.78)	0.148
Altered	18(33.33)	12(44.44)	6(22.22)	
MLLT3				
Unaltered	44(81.48)	19(70.37)	25(92.59)	0.076
Altered	10(18.52)	8(29.63)	2(7.41)	
MTAP				
Unaltered	33(61.11)	14(51.85)	19(70.37)	0.264
Altered	21(38.89)	13(48.15)	8(29.63)	
CDKN2A				
Unaltered	14(25.93)	2(7.41)	12(44.44)	0.004
Altered	40(74.07)	25(92.59)	15(55.56)	
CDKN2B				
Unaltered	22(40.74)	5(18.52)	17(62.96)	0.002
Altered	32(59.26)	22(81.48)	10(37.04)	
ABL1				
Unaltered	51(94.44)	27(100.00)	24(88.89)	0.236
Altered	3(5.56)	0(0.00)	3(11.11)	
NUP214				
Unaltered	39(72.22)	21(77.78)	18(66.67)	0.544
Altered	15(27.78)	6(22.22)	9(33.33)	
PTPN				
Unaltered	40(74.07)	18(66.67)	22(81.48)	0.352
Altered	14(25.93)	9(33.33)	5(18.52)	
LMO1				
Unaltered	31(57.41)	17(62.96)	14(51.85)	0.583
Altered	23(42.59)	10(37.04)	13(48.15)	
LMO2				
Unaltered	50(92.59)	24(88.89)	26(96.30)	0.610
Altered	4(7.41)	3(11.11)	1(3.70)	
CD44				
Unaltered	53(98.15)	27(100.00)	26(96.30)	1.000
Altered	1(1.85)	0(0.00)	1(3.70)	
SLC1A2				
Unaltered	49(90.74)	25(92.56)	24(88.89)	1.000
Altered	5(9.26)	2(7.14)	3(11.11)	
RAG2				
Unaltered	52(96.30)	26(96.30)	26(96.30)	1.000
Altered	2(3.70)	1(3.70)	1(3.70)	
NF1				
Unaltered	47(87.04)	24(88.89)	23(85.19)	1.000
Altered	7(12.96)	3(11.11)	4(14.81)	
SUZ12				
Unaltered	49(90.74)	26(96.30)	23(85.19)	0.351
Altered	5(9.26)	1(3.70)	4(14.81)	
PTPN2				
Unaltered	42(77.78)	22(81.48)	20(74.07)	0.745
Altered	12(22.22)	5(18.52)	7(25.93)	
SH2D1A				
Unaltered	45(83.33)	24(88.89)	21(77.78)	0.467
Altered	9(16.67)	3(11.11)	6(22.22)	
PHF6				
Unaltered	35(64.81)	17(62.96)	18(66.67)	1.000
Altered	19(35.19)	10(37.04)	9(33.33)	
ARHGEF6				
Unaltered	44(81.48)	22(81.48)	22(81.48)	1.000
Altered	10(18.52)	5(18.52)	5(18.52)	

Association of ABD with copy number alterations

The results of Fisher's exact test showed that the ABD group was significantly associated with less *ABL1 *CNA (24% vs 6.35%, p=0.028, Table 4). ABD was not associated with the copy number alterations of genes involved in signaling (*PTEN*, *NF1, *and *PTPN2*, p=1.000, 0.375, 0.612, respectively), cell cycle (*CDKN2A*, *CDKN2B*, and *CASP8AP2*, p=0.344, 0.060 and 0.333, respectively), transcription factors (*LEF1 *and *MYB*, p=1.000, 0.075, respectively), in epigenetic regulator genes (*EZH2*, *SUZ12 *and *PHF6 *(p=0.136, 0.506, 0.473, respectively), with known fusion genes (*STIL*, *TAL1*, *RAG2*, p=0.173, 0.310, 0.490, respectively), and with other genes (*CD44*, *SLC1A2*, *AHI*, *MTAP *and *MLLT3*, and *NUP214*, p=0.490, 0.709, 0.461, 0.207, 0.769, respectively). Further, among immature T-ALL cases, ABD was associated with fewer alterations in *TAL1 *(36.36% vs. 9.30%, p=0.045, Table 5). Similarly, ABD cases exhibited a lower frequency of CNAs in *LMO2 *(27.27% altered vs. 72.73% unaltered). Furthermore, ABD cases exhibited a higher alteration frequency of MYB (54.55% vs. 13.95%, p=0.009). No association of ABD was observed with any of the assessed CNAs in the group of non-immature cases (data not shown).

**Table 4:** Association of absence of biallelic deletion (ABD) with the key copy number alterations in total T-ALL cases

	Total number of patients (%)	ABD absent number of patients (%)	ABD present number of patients (%)	p-value
TAL1				
Unaltered	76(86.36)	56(88.89)	20(80.00)	0.310
Altered	12(13.64)	7(11.11)	5(20.00)	
STIL				
Unaltered	66(75.00)	50(79.37)	16(64.00)	0.173
Altered	22(25.00)	13(20.63)	9(36.00)	
LEF1				
Unaltered	67(76.14)	48(76.19)	19(76.00)	1.000
Altered	21(23.86)	15(23.81)	6(24.00)	
CASP8AP2				
Unaltered	74(84.09)	51(80.95)	23(92.00)	0.333
Altered	14(15.91)	12(19.05)	2(8.00)	
MYB				
Unaltered	71(80.68)	54(85.71)	17(68.00)	0.075
Altered	17(19.32)	9(14.29)	8(32.00)	
AHI1				
Unaltered	78(88.64)	57(90.48)	21(84.00)	0.461
Altered	10(11.36)	6(9.52)	4(16.00)	
EZH2				
Unaltered	60(68.18)	46(73.02)	14(56.00)	0.136
Altered	28(31.82)	17(26.98)	11(44.00)	
MLLT3				
Unaltered	71(80.68)	50(79.37)	21(84.00)	0.769
Altered	17(19.32)	13(20.63)	4(16.00)	
MTAP				
Unaltered	61(69.32)	41(65.08)	20(80.00)	0.207
Altered	27(30.68)	22(34.92)	5(20.00)	
CDKN2A				
Unaltered	35(39.77)	23(36.51)	12(48.00)	0.344
Altered	53(60.23)	40(63.49)	13(52.00)	
CDKN2B				
Unaltered	45(51.14)	28(44.44)	17(68.00)	0.060
Altered	43(48.86)	35(55.56)	8(32.00)	
ABL1				
Unaltered	78(88.64)	59(93.65)	19(76.00)	0.028
Altered	10(11.36)	4(6.35)	6(24.00)	
NUP214				
Unaltered	63(71.59)	46(73.02)	17(68.00)	0.794
Altered	25(28.41)	17(26.98)	8(32.00)	
PTPN				
Unaltered	65(73.86)	46(73.02)	19(76.00)	1.000
Altered	23(26.14)	17(26.98)	6(24.00)	
LMO1				
Unaltered	55(62.50)	40(63.49)	15(60.00)	0.810
Altered	33(37.50)	23(36.51)	10(40.00)	
LMO2				
Unaltered	80(90.91)	59(93.65)	21(84.00)	0.216
Altered	8(9.09)	4(6.35)	4(16.00)	
CD44				
Unaltered	86(97.73)	62(98.41)	24(96.00)	0.490
Altered	2(2.27)	1(1.59)	1(4.00)	
SLC1A2				
Unaltered	79(89.77)	57(90.48)	22(88.00)	0.709
Altered	9(10.23)	6(9.52)	3(12.00)	
RAG2				
Unaltered	86(97.73)	62(98.41)	24(96.00)	0.490
Altered	2(2.27)	1(1.59)	1(4.00)	
NF1				
Unaltered	72(81.82)	53(84.13)	19(76.00)	0.375
Altered	16(18.18)	10(15.87)	6(24.00)	
SUZ12				
Unaltered	75(85.23)	55(87.30)	20(80.00)	0.506
Altered	13(14.77)	8(12.70)	5(20.00)	
PTPN2				
Unaltered	63(71.59)	44(69.84)	19(76.00)	0.612
Altered	25(28.41)	19(30.16	6(24.00)	
SH2D1A				
Unaltered	70(79.55)	51(80.95)	19(76.00)	0.574
Altered	18(20.45)	12(19.05)	6(24.00)	
PHF6				
Unaltered	52(59.09)	39(61.90)	13(52.00)	0.473
Altered	36(40.91)	24(38.10)	12(48.00)	
ARHGEF6				
Unaltered	68(77.27)	48(76.19)	20(80.00)	0.785
Altered	20(22.73)	15(23.81)	5(20.00)	

**Table 5:** Association of absence of biallelic deletion (ABD) with the key copy number alterations in immature T-ALL cases

	Total number of patients (%)	ABD absent number of patients (%)	ABD present number of patients (%)	p-value
TAL1				
Unaltered	46(85.19)	39(90.70)	7(63.64)	0.045
Altered	8(14.81)	4(9.30)	4(36.36)	
STIL				
Unaltered	42(77.78)	35(81.40)	7(63.64)	0.237
Altered	12(22.22)	8(18.60)	4(36.36)	
LEF1				
Unaltered	39(72.22)	33(76.74)	6(54.55)	0.256
Altered	15(27.78)	10(23.26)	5(45.45)	
CASP8AP2				
Unaltered	44(81.48)	34(79.07)	10(90.91)	0.667
Altered	10(18.52)	9(20.93)	1(9.09)	
MYB				
Unaltered	42(77.78)	37(86.05)	5(45.45)	0.009
Altered	12(22.22)	6(13.95)	6(54.55)	
AHI1				
Unaltered	48(88.89)	39(90.70)	9(81.82)	0.590
Altered	6(11.11)	4(9.30)	2(18.18)	
EZH2				
Unaltered	36(66.67)	31(72.09)	5(45.45)	0.150
Altered	18(33.33)	12(27.91)	6(54.55)	
MLLT3				
Unaltered	44(81.48)	36(83.72)	8(72.73)	0.408
Altered	10(18.52)	7(16.28)	3(27.27)	
MTAP				
Unaltered	33(61.11)	26(60.47)	7(63.64)	1.000
Altered	21(38.89)	17(39.53)	4(36.36)	
CDKN2A				
Unaltered	14(25.93)	12(27.91)	2(18.18)	0.708
Altered	40(74.07)	31(72.09)	9(81.82)	
CDKN2B				
Unaltered	22(40.74)	17(39.53)	5(45.45)	0.743
Altered	32(59.26)	26(60.47)	6(54.55)	
ABL1				
Unaltered	51(94.44)	41(95.35)	10(90.91)	0.502
Altered	3(5.56)	2(4.65)	1(9.09)	
NUP214				
Unaltered	39(72.22)	33(76.74)	6(54.55)	0.256
Altered	15(27.78)	10(23.26)	5(45.45)	
PTPN				
Unaltered	40(74.07)	32(74.42)	8(72.73)	1.000
Altered	14(25.93)	11(25.58)	3(27.27)	
LMO1				
Unaltered	31(57.41)	27(62.79)	4(36.36)	0.173
Altered	23(42.59)	16(37.21)	7(63.64)	
LMO2				
Unaltered	50(92.59)	42(97.67)	8(72.73)	0.023
Altered	4(7.41)	1(2.33)	3(27.27)	
CD44				
Unaltered	53(98.15)	43(100.00)	10(90.91)	0.204
Altered	1(1.85)	0(0.00)	1(9.09)	
SLC1A2				
Unaltered	49(90.74)	39(90.70)	10(90.91)	1.000
Altered	5(9.26)	4(9.30)	1(9.09)	
RAG2				
Unaltered	52(96.30)	42(97.67)	10(90.91)	0.369
Altered	2(3.70)	1(2.33)	1(9.09)	
NF1				
Unaltered	47(87.04)	38(88.37)	9(81.82)	0.621
Altered	7(12.96)	5(11.63)	2(18.18)	
SUZ12				
Unaltered	49(90.74)	39(90.70)	10(90.91)	1.000
Altered	5(9.26)	4(9.30)	1(9.09)	
PTPN2				
Unaltered	42(77.78)	34(79.07)	8(72.73)	0.693
Altered	12(22.22)	9(20.93)	3(27.27)	
SH2D1A				
Unaltered	45(83.33)	36(83.72)	9(81.82)	1.000
Altered	9(16.67)	7(16.28)	2(18.18)	
PHF6				
Unaltered	35(64.81)	28(65.12)	7(63.64)	1.000
Altered	19(35.19)	15(34.88)	4(36.36)	
ARHGEF6				
Unaltered	44(81.48)	35(81.40)	9(81.82)	1.000
Altered	10(18.52)	8(18.60)	2(18.18)	

Association of *MEF2C *expression and ABD with *STIL::TAL1* and *NUP214::ABL1* fusions

**Figure 3:**
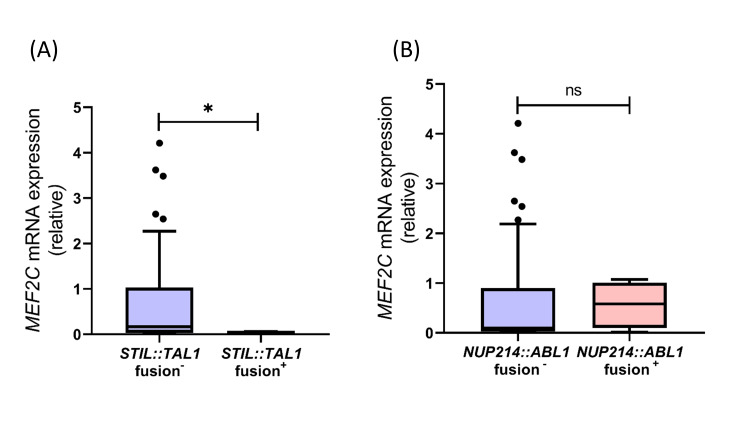
Expression of MEF2C between T-ALL cases with the presence or absence of (A) STIL::TAL1 and (B) NUP214::ABL1 fusion The box plot shows Tukey plots. The Mann-Whitney U test was applied with the level of significance denoted as *ns*, not significant, and *p<0.05.

MLPA-based fusion detection results indicated *STIL::TAL1* fusion was observed in 4 out of 88 T-ALL cases (4.54%). Further analysis revealed that the cases with *STIL::TAL1* gene fusions exhibit lesser expression of *MEF2C *compared to cases without it (p=0.043, Figure 3A). *NUP214::ABL1* fusion was detected in four of the 88 cases (4.54%), however, no difference in *MEF2C *expression was observed between cases with or without *NUP214::ABL1* (p=0.749, Figure 3B). ABD shows no association with either of the analyzed fusions, *STIL::TAL1* and *NUP214::ABL1* (p=1.000 for both, data not shown).

## Discussion

Despite the diversity of genetic abnormalities and gene expression profiles in T-ALL, a limited number of subtypes have been proposed for risk stratification, which is primarily based on distinct expression levels of a few transcription factors. These expression profiles include the *TAL/LMO*, *TLX1*, *TLX3*, and *HOXA* clusters as well as the recently reported proliferative and immature T-ALL subtypes, which are mediated by the overexpression of *NKX2-1*, *NKX2-2*, and *MEF2C*. *MEF2C *functions as a transcriptional regulator, triggering an exhaustive transcriptional program characteristic of early T-cell progenitor (ETP) ALL. Furthermore, the immunophenotypically characterized ETP-ALL overlaps with the immature *MEF2C*-expressing cluster, suggesting that both may represent a single disease entity. The absence of biallelic deletion (ABD) also represents ETP-ALL and is associated with adverse risk features and patients outcome [12,21]. In a previous study, we showed that ABD and high *MEF2C *expression are associated with the immature immunophenotype [12]. Further, we also observed an association of *MEF2C *overexpression with a poor response to prednisolone and MRD positivity after induction, suggesting the use of *MEF2C *expression as a surrogate for true transcriptionally defined ETP-ALL [12]. In this study, we investigated whether ABD and *MEF2C *expression are associated with the common copy number alterations in T-ALL. We observed that copy number alterations in *CDKN2A/B* and *MTAP*, which are otherwise widely established to be associated with cortical/mature immunophenotype, are associated with a low *MEF2C *expression. While we considered it as an obvious association, keeping in mind that *MEF2C *expression is indeed enriched in immature phenotype, our subgroup-specific analysis revealed that the abovementioned association was not present in the immature group but in the non-immature group, thus making a possibility of patient stratification specifically in non-immature T-ALL cases.

Cyclin-dependent kinase inhibitor 2A/B (*CDKN2A/B*) genes at chromosomal arm 9p21 are frequently altered in patients with acute lymphoblastic leukemia (ALL) [22]. Inactivation of these genes by deletion, mutation, and promoter methylation can lead to the malignant transformation of tumor cells and induces chemoresistance in a variety of malignancies [23]. *CDKN2A/2B* and *MTAP *deletion frequency were the highest among the genes analyzed and their codeletion was also previously reported in solid cancers and hematological malignancies [24,25]. In T-ALL, *CDKN2A/2B* frequently exhibits deletion and promoter hypermethylation leading to their downregulation, which is further associated with poor clinical outcomes [26,27]. We and others have previously shown that the frequency of *CDKN2A *deletion was the highest among all gene deletion/duplication evaluated and has variable co-deletion of contiguous genes, including *CDKN2A/B* and *MTAP *present at 9p21.3 cluster [24].

Inactivation of* CDKN2A/B* and other translocations affecting the T-cell receptor genes provide initial insights for genetic defects present in T-ALL. In the context of the biomarker potential, common copy number alterations, such as *CDKN2A/2B***,** are easily detectable in clinics by MLPA. 9p21 deletion and alteration of genes localized on this locus lead to a malignant proliferation of tumor cells and play a crucial role in the pathogenesis and drug resistance of ALL [24,25]. Interestingly, our recent findings also suggested that *CDKN2B *and *MTAP *copy number changes were associated with poor overall patient survival and *CDKN2B *emerged as an independent prognostic factor for poor overall survival and event-free survival [18]. *CDKN2A *alteration has been associated with aberrant *MYC *oncogene activation and novel therapies are under trials to target *MYC*-driven T-ALL [28]. Therefore, considering our observation that patients with *CDKN2A *alteration exhibit low *MEF2C *expression, it will be interesting to investigate whether this association will impact the response to targeted therapy in the future. If any such effect is observed, *MEF2C *expression may also serve as an additional prognosticator. Further, we reported earlier that *MTAP* copy number alterations also act as an independent prognostic factor for patient survival [18]. *MTAP *gene codes for methylthioadenosine phosphorylase, which is involved in purine and methionine utilization metabolism. *MTAP *alterations lead to the deficiency of the salvage pathway for purine synthesis. Some studies suggest that drugs that interfere with purine metabolism, such as methotrexate, might prove beneficial in the treatment of *MTAP* alteration associated with T-ALL. It has been recently shown that *MEF2C *opposes NOTCH-mediated T cell maturation program in the thymus [29]. Therefore, Salt-inducible kinase (SIK) inhibitors, which impair *MEF2C *activity have been proposed to target *MEF2C *expressing ETP-ALL [29]. While SIK inhibitors have been shown to increase prednisolone sensitivity in ETP-ALL cell models, it will be interesting to assess in the future whether this inhibitor can also target a subgroup of patients with non-immature immunophenotype who express high *MEF2C *and does not possess alterations in *CDKN2A/B*.

Interestingly, we also observed the association of ABD with the alteration in the *ABL1* gene, but no association was found with its fusion *NUP214::ABL1*. Activation of the *ABL1 *gene along with the *NUP214::ABL1* fusion is associated with the *TLX1 *and *TLX3 *subgroup [30] while ABD is an established marker of the ETP-ALL subgroup of T-ALL [11]. As we showed in our previous report, ABD and *MEF2C *expression exhibited no association with age, sex, leukocyte count, CNS involvement, and mediastinal mass but with the immature subgroup [12]. ABD shows no association with ETP immunophenotype and prednisolone response while these clinical features were associated with high *MEF2C *expression. In addition to the association of *MEF2C *expression with common copy number alterations, its lower expression in the *STIL::TAL1* fusions was also observed.

This work implies that evaluating *MEF2C *expression along with CNAs may be useful in identifying the patient subgroups with a poor prognosis. However, no positive association of *MEF2C *expression with CNAs was observed, which may limit the translation of this association into the clinics. The fact that this study was conducted in the Indian population may limit its applicability to other populations because there might be variations in the molecular profiles depending on ethnicity. In order to use genetic changes in T-ALL as a risk stratification factor in the clinics, additional research is therefore urgently required.

## Conclusions

The current study demonstrates that low *MEF2C *expression is associated with the alterations of the *CDKN2A/2B* and *MTAP* genes. Taken together, our study demonstrated the association of ABD and *MEF2C *expression with copy number alterations in the *CDKN2A/2B* and *MTAP *cluster in T-ALL. Considering the potential impact of *MTAP *and *CDKN2A/2B* deletion and high *MEF2C *expression on chemosensitivity, it would be interesting to explore the detailed functional role of these associations, which might help address the difficulties in predicting prognosis in this malignancy.
